# Distinction between electron states formed at topological insulator interfaces with the trivial phase and vacuum

**DOI:** 10.1038/s41598-021-91141-2

**Published:** 2021-06-02

**Authors:** A. S. Kazakov, A. V. Galeeva, A. I. Artamkin, A. V. Ikonnikov, L. I. Ryabova, S. A. Dvoretsky, N. N. Mikhailov, M. I. Bannikov, S. N. Danilov, D. R. Khokhlov

**Affiliations:** 1 Physics Department, M.V. Lomonosov Moscow State University, Moscow, 119991 Russia; 2 Chemistry Department, M.V. Lomonosov Moscow State University, Moscow, 119991 Russia; 3 A.V. Rzhanov Institute of Semiconductors Physics, Siberian Branch of RAS, Novosibirsk, 630090 Russia; 4 P.N. Lebedev Physical Institute of RAS, Moscow, 119991 Russia; 5 Faculty of Physics, University of Regensburg, 93053 Regensburg, Germany

**Keywords:** Two-dimensional materials, Topological insulators, Topological insulators, Terahertz optics

## Abstract

In this paper, we show that electron states formed in topological insulators at the interfaces topological phase–trivial phase and topological phase–vacuum may possess different properties. This is demonstrated on an example of heterostructures based on thick topological Hg_1−*x*_Cd_*x*_Te films, in which the *PT*-symmetric terahertz photoconductivity is observed. It is shown that the effect originates from features of the interface topological film–trivial buffer/cap layer. The *PT*-symmetric terahertz photoconductivity is not provided by electron states formed at the interface topological film–vacuum.

## Introduction

Physics of topological insulators (TI) is one of the hottest topics of the modern solid state physics. In these materials, the conduction and valence bands in the bulk are inverted due to strong spin–orbit interaction. Consequently, two-dimensional electron states are necessarily formed in a close proximity of the TI surface. These 2-D electron states possess Dirac dispersion relation with the spin direction locked perpendicularly to the momentum direction^1^. Existence of such topological 2-D electron states was first predicted theoretically^2,3^, and then directly confirmed experimentally through ARPES measurements^4,5^. Topological 2-D electron states are expected to be formed not only at the TI surface, but in heterostructures, at the interface trivial buffer–topological film as well^1,6,7^. It was suggested that the 2-D topological electron states formed at the two types of interfaces mentioned above, have analogous features^1^.

Hg_1−*x*_Cd_*x*_Te solid solutions reveal certain unusual features among topological insulators. HgTe possesses an inverted band structure and represents the TI phase which was unambiguously confirmed by ARPES measurements^8,9^. Increasing the cadmium telluride mole fraction leads to the composition-driven transition from the topological to the trivial insulator phase at *x* = 0.16 (*T* = 0)^10–13^. In contrast to most of the other TIs, Hg_1−*x*_Cd_*x*_Te possess relatively low free carrier concentration^14,15^, which facilitates measurements of electric transport via the topological surface channel on top of the bulk conductivity. Importantly, the same circumstance allows measurements of photoelectric phenomena including photogalvanic effects^16,17^, cyclotron resonance^18–20^ and photoconductivity^21–26^ in the topological phase of Hg_1−*x*_Cd_*x*_Te. In particular, the unusual *PT*-symmetric photoconductivity has been observed in heterostructures based on thick topological Hg_1−*x*_Cd_*x*_Te films, under terahertz excitation^27^.

In the experiments described in^27^, the photoconductivity measurements have been performed at the temperature *T* = 4.2 K in magnetic fields up to 4 T in the Faraday geometry. The photoconductivity amplitude was not an even function of the magnetic field thus breaking the *T* (time reversal) symmetry. It was also different for mirror-symmetric potential probe couples of a Hall bar, demonstrating breaking of the *P* (parity) symmetry. At the same time, simultaneous changing of the magnetic field direction and the potential probe couple kept the photoconductivity intact, revealing therefore the *PT*-symmetry.

The goal of this paper is to reveal the heterostructure element which corresponds to appearance of the *PT*-symmetric photoconductivity. We show that this effect originates from features of the interface between the topological and the trivial phase materials. Instead, it does not appear at the interface topological phase film–vacuum, for which existence of Dirac spin polarized electronic states was confirmed by ARPES previously^8,9^.

## Results

**Figure 1:**
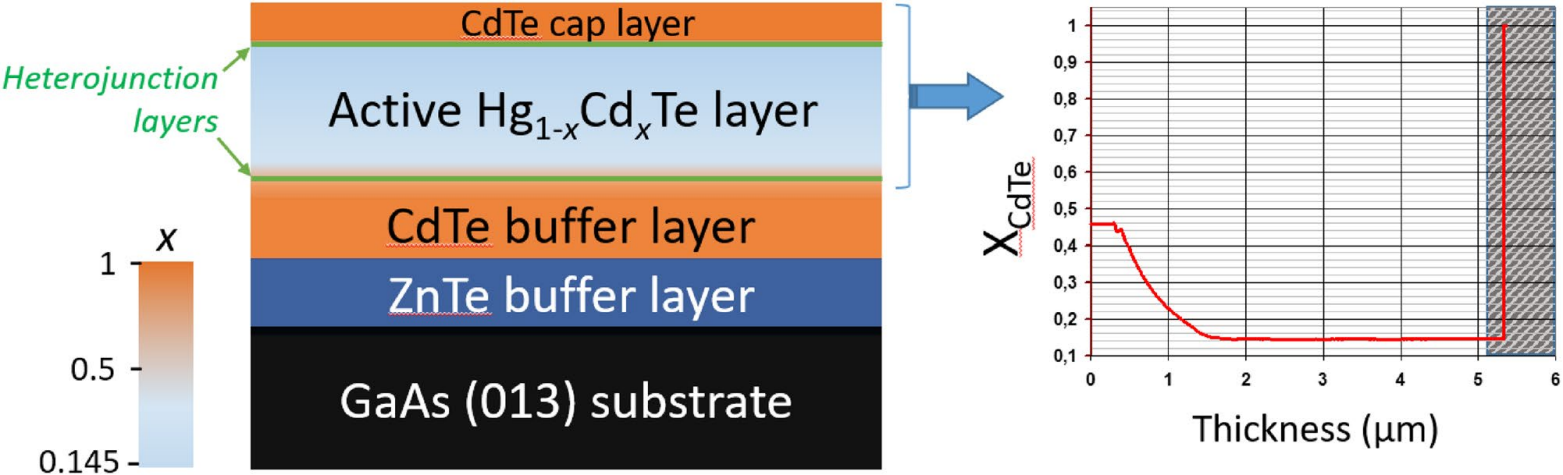
Sequence of layers in heterostructures under study. The graph at the right panel represents the CdTe content *x* distribution in the sample active part. The cap layer indicated by dark gray at the right panel was removed in the etched structure.

We have studied photoconductivity induced by terahertz laser pulses in heterostructures based on 4 μm thick Hg_1−*x*_Cd_*x*_Te films. They were grown on a semi-insulating GaAs substrate via ZnTe and CdTe buffer layers followed by a graded-gap Cd-rich Hg_1−*x*_Cd_*x*_Te relaxed layer. In addition, a protecting 10 nm thick trivial phase CdTe cap layer was deposited on top of the structures (Fig. 1). The cadmium telluride content in the film *x* = 0.145 corresponds to the topological phase. The CdTe content *x* variation at the active layer/trivial buffer interface is smooth, whereas it is sharp at the active layer/cap-layer interface.

To reach the paper goal, two types of structures were studied: the initial one, and the one with the cap layer etched out, so that the Hg_1−*x*_Cd_*x*_Te film surface was open.

Standard Hall bars with the dimensions 5 · 0.5 mm^2^ were prepared by photolithography. Both films were of the *n*-type with the free electron concentration ~ 10^15^ cm^−3^ at the liquid helium temperature. The photoconductivity measurements were performed at *T* = 4.2 K in the Faraday geometry in magnetic fields up to 0.5 T. Further on, we denote the magnetic field direction coinciding with the incident radiation flux as *B*^+^, and the opposite direction as *B*^*−*^.

**Figure 2:**
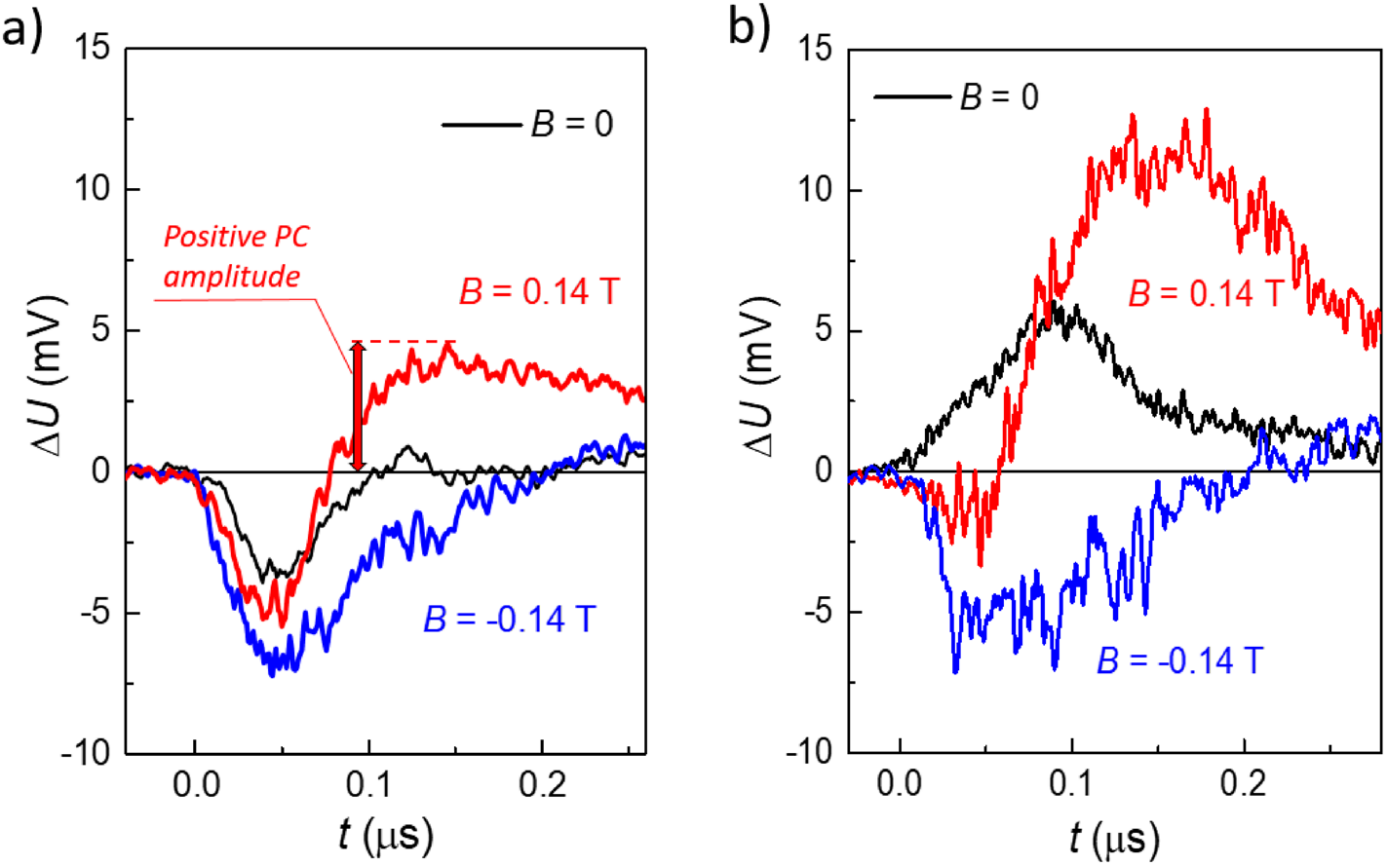
Terahertz photoresponse kinetics measured in the initial (**a**) and etched (**b**) structures in different magnetic fields. For the clarity, the positive *∆U* values in the figure correspond to the positive photoconductivity, the negative *∆U*—to the negative photoconductive response. *B* = 0 (black curves), *B* = 0.14 T (red curves), *B* = − 0.14 T (blue curves). The terahertz radiation frequency *f* = 1.07 THz.

**Figure 3:**
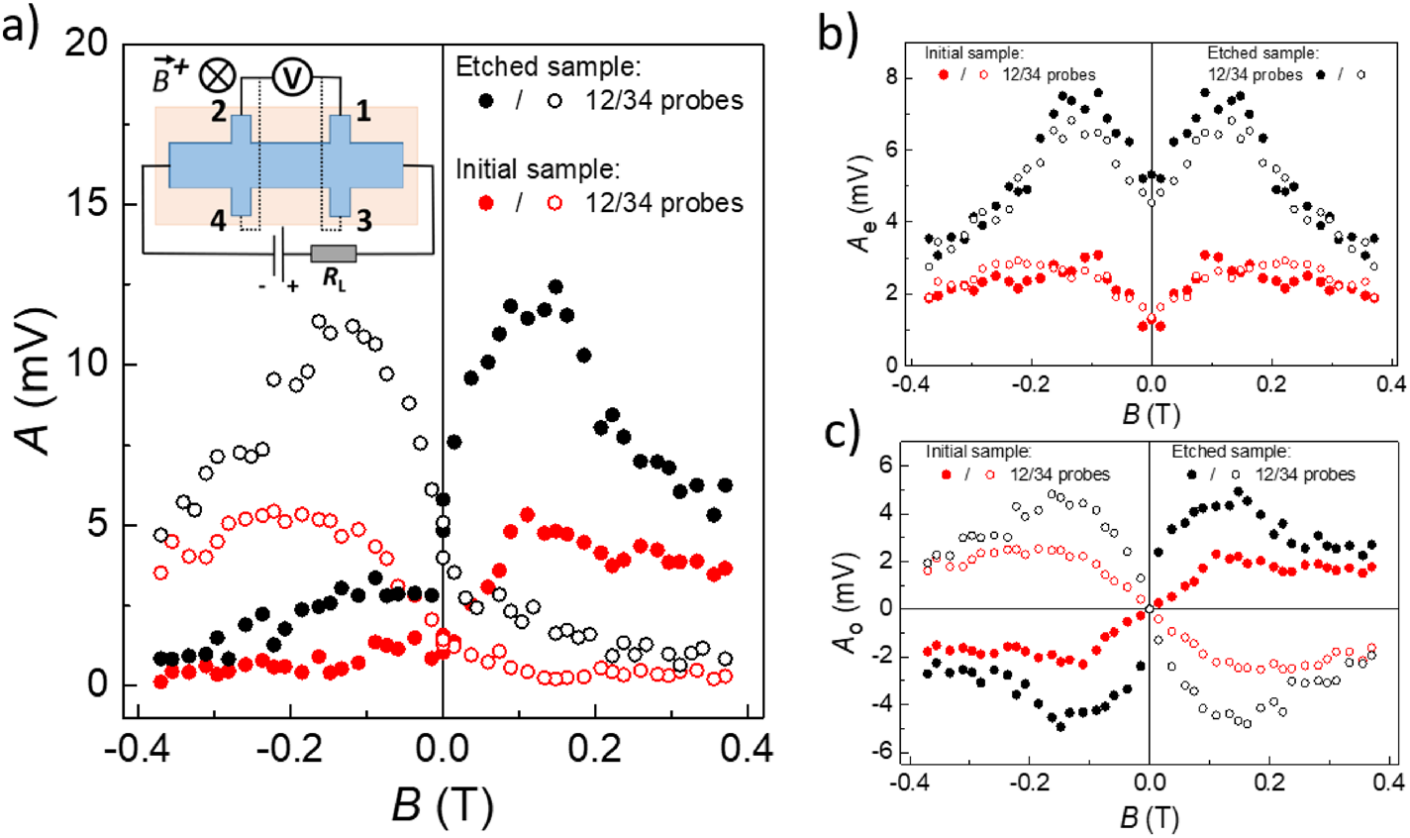
Magnetic field dependence of the positive photoconductivity amplitude. The panel (**a**) shows the magnetic field dependence of the positive photosignal component amplitude. The panels (**b**) and (**c**) represent the magnetic field dependencies of the even and odd parts of the positive photoconductivity, respectively. The measurements were taken between the 1–2 (solid dots) and 3–4 (open dots) potential leads [see the inset in the panel (**a**)] in the initial (red dots) and etched (black dots) structures. Terahertz radiation frequency *f* = 1.07 THz.

Let us consider first the initial structure. The respective photoconductivity kinetics is presented in the Fig. 2a. In the zero magnetic field, the photoconductivity is negative in the beginning of a laser pulse, then it becomes slightly positive. As the magnetic field *B*^+^ grows, the positive photoconductivity is strongly enhanced. Its amplitude *A* (see Fig. 2a for the definition) reaches the maximum at *B*^+^ ≈ 0.15 T and drops in higher fields (Fig. 3a, solid red points). For the opposite magnetic field direction *B*^*−*^, the positive photoconductivity amplitude continuously decreases as the *B*^*−*^ value grows. It should be noted that for the opposite mirror-symmetric couple of potential probes (see the inset in Fig. 3a), the positive photoconductivity behaves in the opposite way: it is enhanced for the *B*^*−*^ field direction, and reduces for the *B*^+^ one (Fig. 3a, open red points). Therefore, the *PT*-symmetric behavior of the positive photoconductivity is observed, as in^27^.

Strikingly, etching out a very thin 10 nm cap layer results in drastic changes of the photoconductivity kinetics and amplitude. Now the photoconductivity is positive in the zero field, the contribution of the negative photoconductivity starts to appear only in non-zero magnetic fields (Fig. 2b). The positive photoconductivity remains *PT*-symmetric, but its amplitude rises by several times compared to the initial structure (Fig. 3, black points).

The key features of the effects observed do not depend on the laser radiation frequency from 1.1 to 3.3 THz, as well as on the radiation polarization, neither linear, nor circular.

## Discussion

The photoconductivity observed in the structures studied consists of several components. The first one is the negative photoconductivity that is almost even in magnetic field and practically does not differ for two mirror-symmetric potential probe couples of a Hall bar. Previously, it was demonstrated that this contribution to the photoconductivity results from heating free electrons by incident THz radiation pulses. Such a heating results in a mobility drop and, consequently, in the negative photoconductivity^25,26^.

The second component is the positive photoconductivity, which is not symmetric neither in magnetic field, nor for the potential probe couples located at the opposite sides of a Hall bar. Phenomenologically, the positive photoconductivity amplitude *A* may be considered as a sum of the even *A*_*e*_ and odd *A*_*o*_ in magnetic field contributions. The respective decomposition of the *A* value into the even *A*_*e*_ and odd *A*_*o*_ components for the two structures studied is presented in the Fig. 3, right upper and lower panels, respectively. It can be seen that while the even in magnetic field contribution *A*_*e*_ is very close for the potential probe couples at the opposite Hall bar sides, the odd contribution *A*_*o*_ for the two Hall bar sides has the same amplitude, but the opposite sign.

Such a decomposition is not just a way to present the results, but has a definite physical meaning. As it was demonstrated in^28^, the odd in magnetic field contribution is due to appearance of the unusual chiral non-local photoconductivity. In the non-local photoconductivity measurement geometry, when the bulk conductivity is excluded, photocurrents induced by the incident terahertz radiation flow along the sample edge around a sample. The photocurrent chirality changes every time the magnetic field direction or the electric bias polarity is reversed. The photocurrents are absent if the magnetic field or the external bias is not applied. Apparently, in the standard Hall bar geometry used in the present study, the chiral non-local photocurrent adds up to the usual bulk photocurrent at one edge of the Hall bar, and is subtracted from it at the opposite edge providing appearance of the odd in magnetic field photoconductivity contribution and giving rise to the *PT*-symmetric photoconductivity.

The symmetric in magnetic field photoconductivity part *A*_*e*_ may have a traditional origin and correspond to photoexcitation in the sample bulk. The answer to the question on what is the heterostructure element that is responsible for appearance of the odd in magnetic field photoconductivity component *A*_*o*_, is not so straightforward.

**Figure 4:**
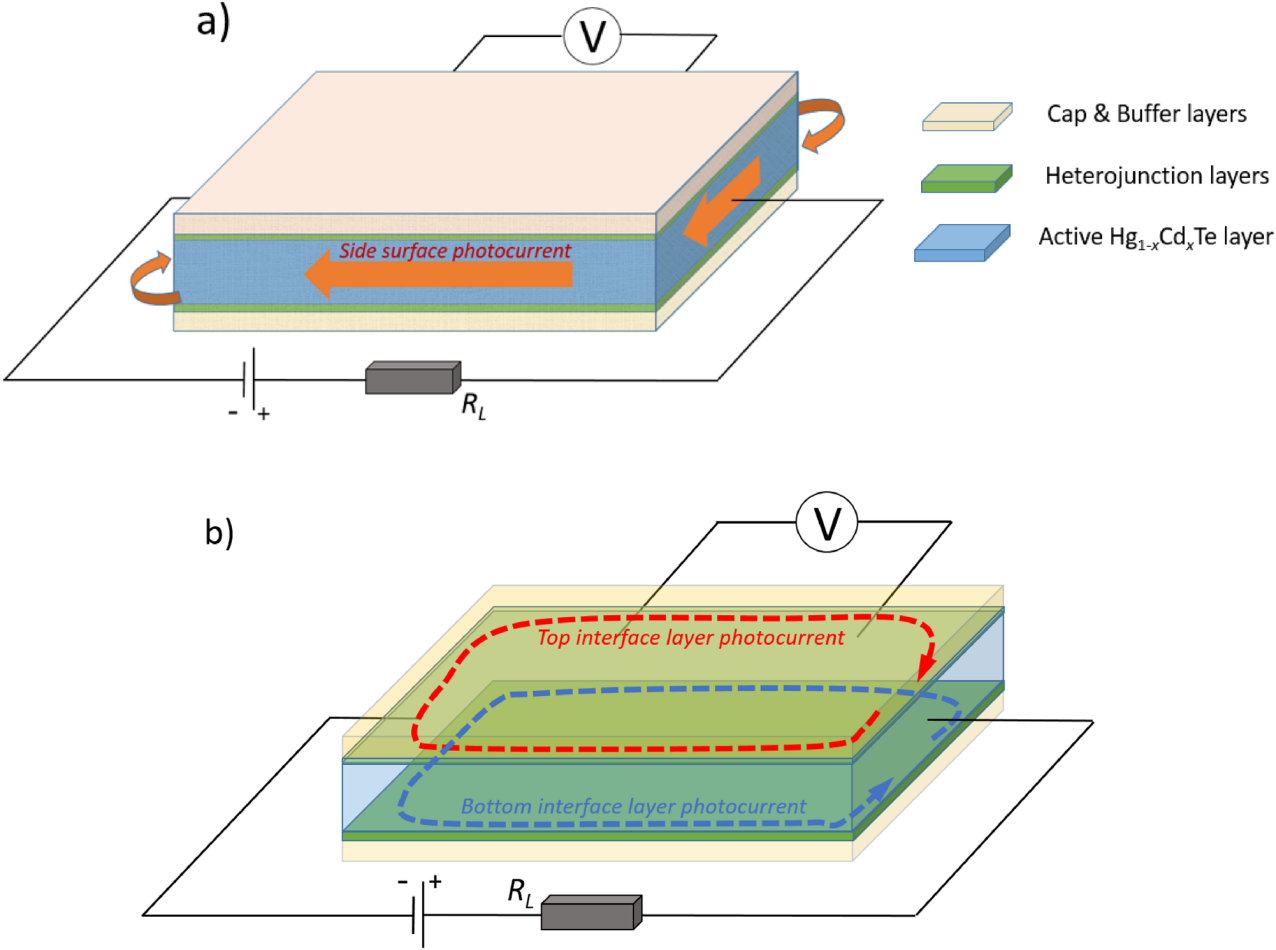
Possible options for the element of the structure responsible for the odd photoconductivity component appearance. (**a**) The chiral photocurrent flows along the topological film–vacuum interface; (**b**) chiral photocurrents flow along the edges of the topological film–trivial buffer/cap-layer interfaces.

First, it is clear that the bulk of the film may not be such an element since the respective photocurrent flows along a sample edge. Therefore, only the side surface of the film and the edges of the film–buffer layer and film–cap layer interfaces may be candidates for this element (Fig. 4). Let us consider these options.

If we suppose that the *A*_*o*_ photocurrent flows along the side film surface representing the topological film–vacuum interface (Fig. 4a), then etching out the cap layer would lead to disappearance of the effect. Indeed, after removal of the cap layer, the top film surface becomes the topological film–vacuum interface as well. Then the respective photocurrent would flow not only along the sample edge, but via the top surface, too, thus shunting the edge photocurrent contribution. Experimentally, however, the *PT*-symmetric photoconductivity is strongly enhanced upon removal of the cap layer (see Fig. 3), to the contrary of the expected behavior. Therefore, the topological film–trivial buffer and topological film–trivial cap layer interfaces are the only remaining options.

The second important point is the following. Apparently, the *A*_*o*_ photocurrent components corresponding to the film–buffer and film–cap layer interfaces, to a great extent compensate each other (Fig. 4b). Indeed, when the film–cap layer interface disappears in the etched structure, the antisymmetric photocurrent component *A*_*o*_ grows by several times (see Fig. 3). The reason for such a behavior may be the following. As it was suggested in^26^, the positive photoconductivity in the zero magnetic field is due to diffusion of electrons heated in the film bulk, to the heterojunction area, where the topological layer is located. When entering the topological layer, the diffusing electrons acquire higher mobility thus providing appearance of the positive photoconductivity. In the non-zero magnetic field, such a diffusion occurs along or against the magnetic field direction for the two different interfaces. This could be a reason for the fact that the two interfaces act towards each other and compensate the effect significantly. The effect amplitude apparently depends on the details of the interface structure, so the film–buffer and the film–cap layer interfaces do not compensate each other exactly.

As it has been shown in^19^ for analogous structures based on thick topological Hg_1−*x*_Cd_*x*_Te films, the electronic states located at smooth and sharp interfaces between the topological and trivial phases have different structures. While the sharp interface forms 2D electron states, the smooth interface produces 3D-like states. It was suggested that the sharp interface forms true 2D electron states, whereas for the smooth interface, a series of Volkov–Pankratov interface states are formed^19^. In the latter case, these electronic states are effectively 3D, in an analogy to conventional quasi-2D electron states in a wide quantum well occupying multiple subbands.

The results obtained in the present study suggest that both true 2D interface states at the topological film–cap layer interface and quasi-3D interface states at the topological film–trivial buffer interface provide appearance of the chiral non-local edge photocurrents and, consequently, of the *PT*-symmetric photoconductivity. Moreover, it seems that the effect is more strongly pronounced for the smooth interface since the amplitude does not change sign upon removal of the cap layer. The reasons for such a behavior may be the following.

The Volkov–Pankratov interface states, while being quasi-3D, are located at the interface and still possess higher mobility compared to the film bulk, which is an important condition for appearance of the positive photoconductivity in the zero magnetic field. Another possible option is related to the alloy composition fluctuations at the smooth interface. In such a situation, the 2D topological state may be not flat, as it is for the sharp interface, but rippled. Then the effective surface of the 2D boundary between the TI film and the trivial buffer at the smooth interface may be larger than for the sharp interface between the film and the cap layer providing higher chiral photocurrent.

It should be stressed that the microscopic origin of the chiral non-local edge photocurrents providing appearance of the *PT*-symmetric photoconductivity is still unclear. Moreover, it contradicts apparent symmetry arguments^27,28^. It means that there exists an unaccounted external factor that breaks the *P*- and *T*-symmetries and leads to the effects observed. The origin of this external factor is to be determined, it is only clear that it is not a deviation from the ideal experimental geometry or any kind of the sample anisotropy^27^. Any sort of a microscopic model for the effect origin must take into account this factor.

On the other hand, the results presented show that the interface between the topological film and the trivial buffer/cap layer is the heterostructure element where the effect appears. The topological film–vacuum interface does not possess such a feature.

## Conclusions

In summary, we have demonstrated that the electron states formed at the interfaces topological insulator–trivial insulator and topological insulator–vacuum possess different features. In particular, we have shown that the *PT*-symmetric terahertz photoconductivity observed in heterostructures based on thick Hg_1−*x*_Cd_*x*_Te films being in the topological phase, results from processes at the interfaces film–buffer/cap layer, while the interface film–vacuum does not possess this feature. Besides, it was shown that the topological phase–trivial phase buffer and the topological phase–trivial phase cap layer form interfaces that strongly compensate each other in the effect of *PT*-symmetric photoconductivity. Finally, the *PT*-symmetric photoconductivity effect was directly linked to the non-local chiral terahertz photoconductivity. A way to extract the contribution of the chiral non-local photoconductivity from measurements in the traditional Hall bar geometry has been presented.

## Methods

The samples were grown by the MBE technique on semi-insulating $\left\langle {013} \right\rangle$ GaAs substrates. Composition of all layers of the initial heterostructure was controlled by ellipsometry in situ during growth^29^. The accuracy of the composition *x* measurements was about 0.001, as estimated through the ellipsometric results. The procedure accuracy was confirmed by measurements of transmission spectra in samples with successively etched surface layers^30^. The active layer composition *x* = 0.145 in the initial heterostructure corresponds to the topological phase.

In the second heterostructure studied, the cap CdTe layer was removed using the HBr + 0.01% Br_2_ etchant. The etching rate ~ 0.05 μm/min was controlled by measuring the shift of interference peaks in the transmission spectra of the initial and the etched control sample. Deletion of the CdTe cap layer was monitored through measurements of the reflectivity spectra. The whole process was performed analogously to described in^31,32^.

**Figure 5:**
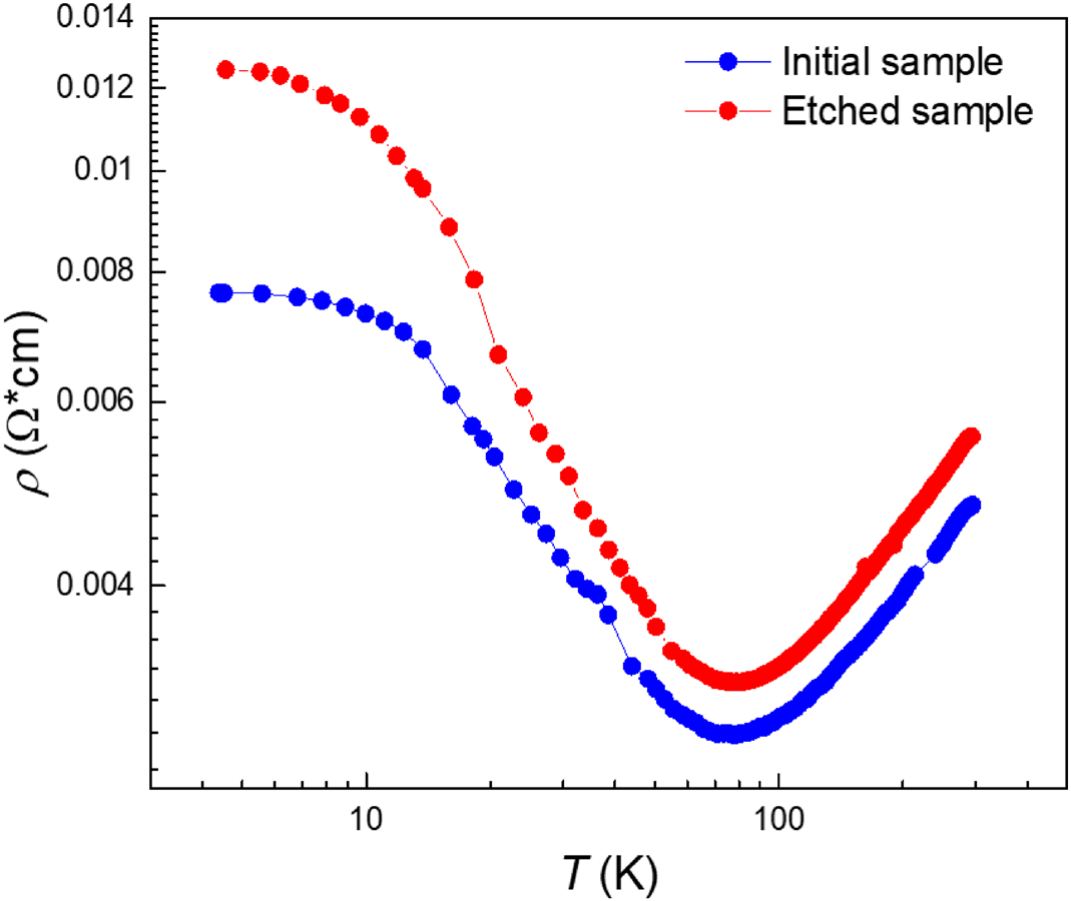
Resistivity temperature dependence of the samples under study.

Electrical characterization of the samples has been done in the temperature interval 4.2–300 K. The resistivity temperature dependence of the samples studied is shown in the Fig. 5. This dependence has a minimum which is characteristic for the inverted band structure of Hg_1−*x*_Cd_*x*_Te, and hence the topological phase^13,25^.

The photoconductivity was excited by a pulsed NH_3_ gas laser with the pulse length ~ 100 ns. The laser radiation frequency was 1.1 or 3.3 THz with the power in a pulse up to 10 kW. The beam had an almost Gaussian profile with the spot size 3–5 mm depending on the laser frequency. The radiation polarization, either linear or circular, was controlled by rotation of λ/2 or λ/4 plates introduced into the laser beam. More details on the experimental setup may be found in^33–36^.

The sample was directly immersed in the liquid helium to minimize possible sample heating effects. The magnetic field up to 0.5 T, if applied, was directed perpendicularly to the sample surface. The photoconductivity measurements were performed using the 4-probe method, for two opposite directions of the applied bias, as well as with the zero bias to control a possible appearance of the photovoltaic signal. In all cases, the photovoltaic contribution to the signal was negligible.
